# Comment on “An Informatics Approach to Evaluating Combined Chemical Exposures from Consumer Products: A Case Study of Asthma-Associated Chemicals and Potential Endocrine Disruptors”

**DOI:** 10.1289/EHP447

**Published:** 2016-09-01

**Authors:** Anne Steinemann

**Affiliations:** The University of Melbourne, Parkville, Victoria, Australia

Gabb and Blake put forth a database to assess potential exposure risk to 55 asthma-associated and endocrine-disrupting chemicals in a range of 38,975 consumer products. The foundation of their database is publicly available ingredient information. While the authors acknowledge that products may not disclose all ingredients, they did not acknowledge the potential magnitude of that nondisclosure and the impact on the credibility and utility of the database. For example, a recent gas chromatography–mass spectrometry (GC-MS) analysis of common fragranced consumer products, similar to those in the database, found that more than 97% of product ingredients were completely undisclosed (Steinemann 2015). Each product’s undisclosed ingredients included at least 1 of the authors’ 55 target chemicals.

The authors note that the Fair Packaging and Labeling Act (FPLA) requires manufacturers to list ingredients in descending order of predominance, except that fragrance ingredients can be listed simply as “fragrance.” However, this requirement of the FPLA does not apply to all database products. About 2,000 of the products, most of them in the “household” category, fall under the regulation of the Consumer Product Safety Act (CPSA). The CPSA does not require the listing of specific product ingredients or the listing of the general term “fragrance” (Steinemann et al. 2011).

Indeed, in the aforementioned GC-MS analysis (Steinemann 2015), more than two-thirds of the tested fragranced consumer products that were regulated under the CPSA did not disclose fragrance on the label. Further, none of the tested fragranced consumer products that were regulated under any U.S. law disclosed all fragrance ingredients. The most common and undisclosed fragrance ingredients (e.g., limonene, α-pinene, and β-pinene) are included among the authors’ 55 target chemicals.

The authors’ database reported target chemicals in 15.3% of the air fresheners, 5.5% of the cleaners, 14.2% of the dishwashing products, and 3.3% of the laundry products assessed, based on publicly available ingredients. In contrast, the GC-MS analysis (Steinemann 2015) found target chemicals in 100% of the air fresheners, 100% of the cleaners, 100% of the dishwashing products, and 100% of the fragranced laundry products assessed. This discrepancy is likely due to the facts that *1*) products are not required to disclose all ingredients, *2*) products are not required to disclose the term “fragrance,” and *3*) the authors did not perform GC-MS analyses on the products in their database.

I raise these concerns because the authors’ database may provide a significantly underestimated risk assessment and false assurance to consumers because of its sole reliance on publicly reported data—which can represent only a small percentage of all ingredients in consumer products.

## References

[r1] Steinemann A 2015 Volatile emissions from common consumer products. Air Qual Atmos Health 8 3 273 281, doi:10.1007/s11869-015-0327-6

[r2] Steinemann AC MacGregor IC Gordon SM Gallagher LG Davis AL Ribeiro DS 2011 Fragranced consumer products: chemicals emitted, ingredients unlisted. Environ Impact Assess Rev 31 3 328 333, doi:10.1016/j.eiar.2010.08.002

